# The PediPERForm Learning Network congenital perfusion registry

**DOI:** 10.1051/ject/2024037

**Published:** 2025-06-16

**Authors:** Brian L. Mejak, James A. Reagor, Vince F. Olshove, Donald S. Likosky

**Affiliations:** 1 Department of Perfusion, Children’s Hospital Colorado Aurora Colorado 80045 USA; 2 Department of Cardiovascular Perfusion, Cincinnati Children’s Hospital Medical Center and Department of Pediatrics, University of Cincinnati College of Medicine Cincinnati Ohio 45229 USA; 3 Department of Perfusion, Norton Children’s Hospital Louisville Kentucky 40202 USA; 4 Department of Cardiac Surgery, Frankel Cardiovascular Center, Michigan Medicine, University of Michigan Ann Arbor Michigan 48109 USA

**Keywords:** Congenital, Perfusion, Registry, PediPERForm

## Abstract

Medical procedural registries are uniquely positioned to support shared decision-making through risk prediction modeling, support quality assessment and improvement through performance benchmarking, and provide public reporting of evidence-based practices and outcomes. For example, the Centers for Disease Control and Prevention (CDC) consulted the Extracorporeal Life Support Organization (ELSO) registry to assess the severity of the swine flu outbreak in 2009–2010. The development and growth of The Society of Thoracic Surgeons Congenital Heart Surgery Database (STS-CHSD) has positively contributed to the congenital heart surgery community by developing objective mortality STAT categories and complexity stratification for operations, a common nomenclature for classifying operations and reporting the costs associated with complications for nine benchmark operations. Within the setting of adult cardiac surgery, the Perfusion Down Under Collaborative has used its registry to develop quality improvement initiatives, including those related to the management of arterial outlet temperature, glucose, and arterial pCO_2_. The PERForm registry leverages data from nearly 50 US hospitals to support targeted quality improvement initiatives within the setting of adult cardiac surgery. The PERForm registry participants receive benchmark reports and participate in quarterly collaborative learning meetings noted for unblinding hospital performance data. In 2014, with no current congenital cardiopulmonary bypass (CPB) registries, various experts within the congenital perfusion community and leaders from the PERForm registry began working to develop a pediatric perfusion registry. From this work, the PediPERForm Learning Network (PLN) and its associated congenital perfusion registry became active and began collecting data in October 2021.

## Overview

Medical procedural registries are uniquely positioned to support shared decision-making through risk prediction modeling, support quality assessment and improvement through performance benchmarking, and provide public reporting of evidence-based practices and outcomes. For example, the Centers for Disease Control and Prevention (CDC, Atlanta, Georgia) consulted the Extracorporeal Life Support Organization (ELSO, Ann Arbor, Michigan) registry to assess the severity of the swine flu outbreak in 2009–2010. The development and growth of The Society of Thoracic Surgeons Congenital Heart Surgery Database (STS-CHSD) has positively contributed to the congenital heart surgery community by developing objective mortality STAT categories and complexity stratification for operations, a common nomenclature for classifying operations [1], and reporting the costs associated with complications for nine benchmark operations [2]. Within the setting of adult cardiac surgery, the Perfusion Down Under Collaborative has used its registry to develop quality improvement initiatives, including those related to the management of arterial outlet temperature, glucose, and arterial pCO_2_ [3]. The PERForm registry leverages data from nearly 50 United States (US) hospitals to support targeted quality improvement initiatives within the setting of adult cardiac surgery [4]. The PERForm registry participants receive benchmark reports and participate in quarterly collaborative learning meetings noted for unblinding hospital performance data [5].

In 2014, with no current congenital cardiopulmonary bypass (CPB) registries, various experts within the congenital perfusion community and leaders from the PERForm registry began working to develop a pediatric perfusion registry. From this work, the PediPERForm Learning Network (PLN) and its associated congenital perfusion registry became active. They began collecting data in October 2021 where real time data and clinical questions can be addressed immediately. PLN became the American Society of Extracorporeal Technology’s official societal partner in 2021 which facilitates cooperation and professional development between the two organizations.

## Description

A workgroup of 10–20 congenital perfusionists across the US, Europe, and Australia began regularly set conference calls to develop the congenital registry and decided on six areas of focus: Anticoagulation, equipment usage and circuit selection, fluid balance, prime constituents, laboratory values (e.g., hematocrit, lactates, and creatinine), and blood product usage (Table 1). Field definitions and data specifications were defined and agreed upon by the workgroup. Furthermore, when applicable, the definitions were harmonized with the STS-CHSD and Adult PERForm registries. Over 130 data definitions were documented. Subsequently, the data collection instrument was tested at four pediatric centers within the workgroup, iteratively enhancing the rigor of our approach through feedback from our contributing members.

**Table 1:** Data fields collected on every case.

Gender	VAVD use	Post heparin ACT
Weight	VAVD Measurement Location	Post Protamine ACT
Height	Max VAVD	First ICU Creatinine
Fundamental Diagnosis	ANH use	Baseline Creatinine
Admission Date	ANH Volume	Post-op Max 48hr Creatinine
Arterial Cannula	RAP/VAP use	Baseline Hematocrit
Arterial Cannula Type	RAP/VAP Volume	First Hematocrit in OR
Arterial Cannula Size	RAP/VAP Volume Returned	Lowest Hematocrit on CPB
Arterial Filter Type	24 Hour Chest Tube Output	First Hematocrit in ICU
Arterial Line Size	Residual Volume Processed	Last Hematocrit on CPB
Arterial Filter Pore Size	Residual Volume Returned	Last Hematocrit pre CPB
Arterial Pump Type	Blood Products Used	Post Protamine Hematocrit
Venous Cannula Manufacturer	Cryo Volume on CPB	Heparin Dose Response
Venous Cannula Style	FFP Volume on CPB	Heparin Concentration Used
Venous Cannula Size	Platelet Volume on CPB	Maximum HPT on CPB
Venous Line Size	PRBC Volume on CPB	Minimum HPT on CPB
ATS System Used	Whole Blood Volume on CPB	Post Heparin HPT
Biocoating Used	Cryo Volume off CPB	Post Protamine HPT
Biocoating Name	FFP volume off CPB	First Lactate in OR
Hemoconcentrator Type	Platelet Volume off CPB	Last Lactate pre CPB
Oxygenator Type	PRBC Volume off CPB	First Lactate on CPB
Pump Boot Size	Whole Blood off CPB	Last Lactate on CPB
Venous Reservoir Type	Cryo Volume in Prime	Post Protamine Lactate
Prime Fluids	FFP Volume in Prime	24 Hour Maximum Lactate
Prime Fluid Volumes	Platelet Volume in Prime	Age at DOS
Prime Treatment	PRBC Volume in Prime	Surgeon Name
Prime Medications Used	Whole Blood Volume in Prime	Primary Perfusionist ID
Prime Medication Name	Urine Volume on CPB	Primary Perfusionist Name
Prime Medication Dose	Modified Ultrafiltration Used	Primary Perfusionist Agency
Prime Medication Volume	Modified Ultrafiltration Time	Assistant Perfusionist ID
Prime Volume	Modified Ultrafiltration Type	Assistant Perfusionist Agency
Highest Arterial Line Temp.	MUF Volume Removed	Assistant Perfusionist Name
Lowest Temperature on CPB	Ultrafiltration Used	Assistant Perfusionist Agency
Lowest Temperature Source	ZBUF/DUF Used	Perfusion Student
Temp at Separation from CPB	Ultrafiltration Volume	Student Perfusionist Name
Cardioplegia System	Residual Pump Volume	Anesthesiologist Name
Cardioplegia Solution	Irrigation Solution Volume	Reoperation
Cardioplegia Blood Ratio	Wall Waste Volume	CPB Time
Cardioplegia Crystalloid Ratio	ATS Volume Collected	DHCA Time
Cardioplegia Total Volume	ATS Volume Returned	Cerebral Perfusion Used
Cardioplegia Crystalloid Volume	Crystalloid Volume on CPB	Cerebral Perfusion Time
ph Management	Colloid Volume	Cross Clamp Time
ph Management Temp Source	Medication Volume on CPB	Diagnosis
ph Stat During Cooling	Fluid Balance	Primary Diagnosis
ph Stat Cooling Threshold	Baseline ACT	Procedure
Alpha Stat During Warming	Maximum ACT on CPB	Primary Procedure
Alpha Stat Warming Threshold	Minimum ACT on CPB	Intraoperative Death

To support the interdisciplinary nature of the intended work, facilitate ease of implementation, reduce start-up costs, and take advantage of a great deal of experience and expertise [6], the decision was made to partner with the Pediatric Cardiac Critical Care Consortium (PC^4^) under the collaboration umbrella of Cardiac Networks United (CNU). PC^4^ was instrumental to the development of PLN by providing expert opinions on PLN’s framework, sharing Institutional Review Boards (IRBs), and sharing of data managers and data management. This strategic partnership also allows for sharing of longitudinal data across registries through established regulatory pathways at participating institutions.

PLN governance consists of three organizational committees. The Executive Committee comprises at least seven members, including the PLN Co-Directors, the Scientific Review (SRC) and Quality Improvement (QIC) committee chairs, and three at-large members. Duties of the Executive Committee include general oversight and strategic direction of the network, the development, maintenance, and optimization of organizational partnerships, minimization of redundancy and data integration across CNU registries, periodic evaluation, and modification of data fields and definitions, providing the SRC and QIC committees with guidance and direction, and assist the SRC and QIC decision making when necessary and appropriate.

PLN’s SRC consists of the SRC Chair, one representative from AmSECT’s Quality Improvement Committee, one representative from AmSECT’s Pediatric and Congenital Perfusion Committee, and two to four at large members from PLN participating centers. Duties of the SRC include review, acceptance, or feedback to data and research requests utilizing PLN data.

The QIC consists of standing members, including the QIC Chair, representation from AmSECT’s Quality Improvement Committee, representation from AmSECT’s Pediatric and Congenital Perfusion Committee, and one *ad hoc* representative from each member institution. Duties of the QIC include identifying variations in practice, development, and dissemination of quality improvement projects aimed to improve care during pediatric and congenital CPB.

Data may be entered into the PLN registry using three software solutions. CardioAccess (Fort Lauderdale, Florida) and Lumedx (Interlad Medical Systems, Montreal, Quebec) are two software programs designed for data collection for the STS and other cardiac-related registries. CardioAccess and Lumedx have PLN modules and extract data fields from other modules in the STS software. Alternatively, a hospital may develop its data entry software. All three options require software validation by PLN leadership. PLN records are then submitted to Arbormetrix’s (Ann Arbor, Michigan) live reporting dashboard within one month of the date of surgery. The reporting platform is separated into eight categories: Equipment, Blood Products, Prime, Fluid Management, Lab Parameters, Case Mix, Cardiopulmonary Bypass, and Data Quality. These categories are further broken down into Blinded Site Comparison, Perfusionist Comparison, and Institutional reports within the Arbormetrix live dashboard (Table 2).

**Table 2:** Reporting measures.

Case Volume	Arterial Line Type and Size	Circulatory Arrest Time
Oxygenators	Venous Reservoir Type	ANH Volume
Arterial Line Size	Blood and Blood Products	Fluid Balance
Venous Line Size	Prime Volume	Urine Output
Pump Boot Size	Albumin Use in Prime	ATS Volume Transfused
Circuit Coating	Amicar Use in Prime	Change in Hematocrit
Hemoconcentrator	Tranexamic acid Use in Prime	Last Hematocrit on CPB
Arterial Pump Head Type	Antibiotic Use in Prime	Post-Protamine Hematocrit
ATS	Autologous Circuit Prime Volume	Last Lactate on CPB
Arterial Cannulas	Bypass Time	Change in Lactate
Venous Cannulas	Cross-clamp Time	Target HPT
Cannula Size	Regional Perfusion Time	

To maintain data privacy, the Blinded Site Comparison report enables authorized users to identify their centers, with the identification of other centers masked through a centrally maintained and secured blinded identifier. The Institutional reports allow participating centers to evaluate trends in their data and in the Perfusionist Comparison reports, centers may view their institutions’ perfusionist data too. Reports and fields may be filtered by the surgeon, perfusionist, anesthesiologist, date range, six age categories, eight weight groups, five STAT rankings, primary benchmark procedures, deep hypothermic circulatory arrest use, and five lengths of bypass time periods (Table 3). The data is harvested monthly from Arbormetrix to the PC^4^ Data Coordinating Center at the University of Michigan. Requests for data interpretation and statistical analysis for research and quality projects are performed by PLN’s data coordinators.

**Table 3:** Filter categories.

Weight groups	Age categories	Length of CPB
All	All	All
0–3 kg	Neonate <30 days	<1 h
3–6 kg	Infant = >30 to <365 days	1–2 h
6–10 kg	Toddler = >1 to 5 years	2–3 h
10–20 kg	Child = >5 to 13 years	3–4 h
20–40 kg	Teenager = >13 to 18 years	4+ h
40–60 kg	Adult = >18 years	

By design, each participating institution designates a clinical champion tasked with managing their center’s data entry and accuracy, facilitating all regulatory and contractual matters, and coordinating mandatory audit procedures at their institution. This clinical champion also provides their center with informative data such as benchmarking information comparing their site to the rest of PLN and internal performance data. It handles personnel changes in the registry fields. Institutional members must also be active members in PC^4^ (contracting with the University of Michigan, PLN is considered a module of PC^4^) and obtain IRB approval or exemption prior to data submission. Adherence to these participation requirements allows the institution access to benchmarking data, structured reports, real-time institutional and PLN dashboards, and data requests for scientific studies and quality improvement initiatives.

To ensure data accuracy and completeness, data audits are conducted within 9 months, but no later than 1 year after a site’s data collection begins. The auditor is one of the PLN Executive Committee members. Initial site audits are done in person, with additional days of remote audits as necessary until the audit is complete. Before the audit, the data coordinating center (DCC) will assess records in the PLN registry for missing data against the site’s PC^4^ records. Within the date range selected for the audit, all PC^4^ records with a surgical procedure requiring CPB should have an accompanying PLN record. Once all appropriate records are verified as existing in the registry, the DCC generates a randomly selected set of PLN records, including 30 records or a 10% sample size for sites with smaller case volumes. The auditor then works with the site’s clinical champion to review the records for accuracy and completeness. As all fields are mandatory for submission, all fields are audited for each record. Utilizing an ‘over the shoulder’ approach, the clinical champion opens the EMR for selected cases and reports a value for each pre-selected data variables. A score sheet, formatted by the PLN DCC, is used to record the findings. Discrepancies are identified and resolved in real time. These discrepancies are logged into a report. Following the audit, a wrap-up meeting occurs with the auditor and site clinical champion to review the findings. After reviewing the audit reports, the score sheet is returned to the DDC where major and minor discrepancies and overall accuracy rates are calculated. Major and minor discrepancies were voted on by members of the executive committee. Upon completion, results are sent to the clinical champion along with an audit findings report.

Subsequent audits are conducted once every 3 years. The subsequent audit format is identical to the initial audit with the only exception being audits may be held entirely remotely, unless there has been significant turnover in the site’s perfusion team, clinical champion, or data abstractors. Once an audit report has been issued to the site, including all findings, affected records, recommendations, requirements for resolution of any discrepancies, the site’s plan for corrective actions and any necessary data cleaning should be submitted to PLN with a timeline for completion of the corrective actions.

To ensure the highest level of data quality for all quality improvement (QI) processes, quality assurance (QA), or research requests utilizing PLN registry data, discrepant findings during a site data audit with a major discrepancy rate (MDR) in excess of 5% and/or an overall accuracy (OA) less than 90% will be considered an audit failure. Should a site fail an audit, the site’s data will be quarantined from use for any QA, QI, or research project. Once the data has been cleaned and updated and any systematic issues resolved, the quarantine will be lifted. An MDR of less than 5% or an OA of greater than 90% is considered a successful audit, and the site’s data may be used in QA, QI, and research projects. However, the site must correct any data errors and systematic issues identified during the audit.

No QI, QA, or research projects have been completed since the inception of data submission in October of 2021. The executive committee decided to wait on all data requests until site audits were complete and validated and a significant amount of record submissions were present in the registry.

**Figure 1:**
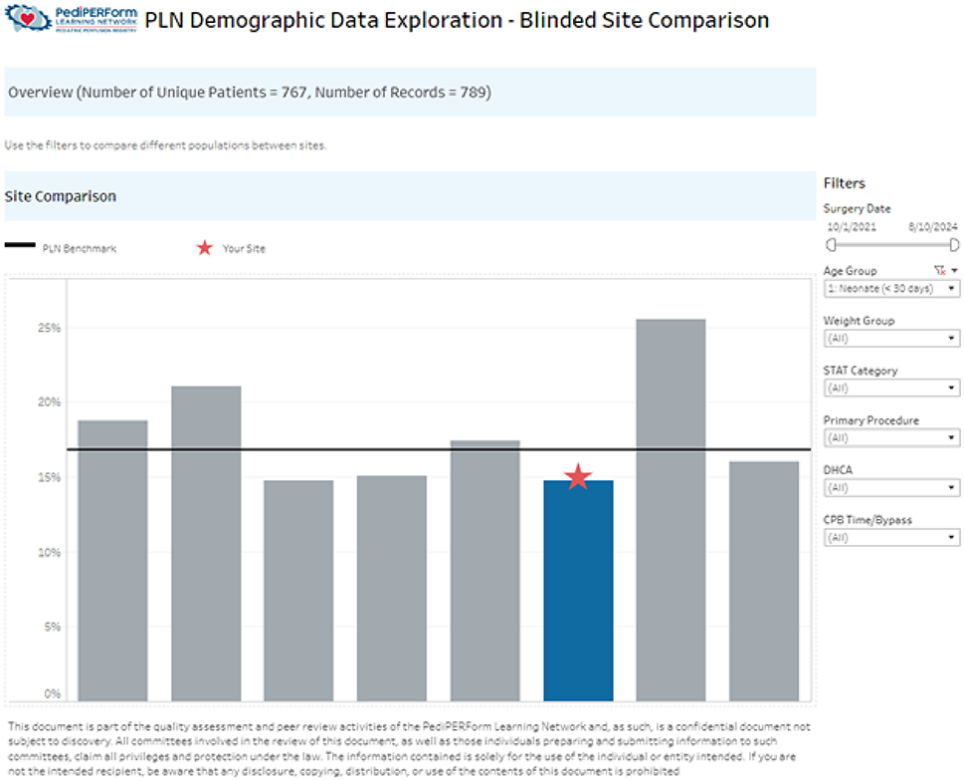
Blinded site comparison report showing number of neonatal CPB cases.

PLN’s reporting platform provides three main categories of data visualizations including blinded, institutional, and perfusionist comparison reports (Table 2). The blinded reports return aggregate data for each site. The sites are blinded by way of a site ID which changes each year. The site ID is a randomly generated number that changes at the first of the year, which is shown on comparison graphs and data. To facilitate learning and sharing, the clinical champion at each site has access to the blinded site key. This allows clinical champions to communicate about techniques, technologies, device use, and other topics. Data in blinded site reports is aggregate data only and cannot be separated out into specific case data (Figure 1).

**Figure 2:**
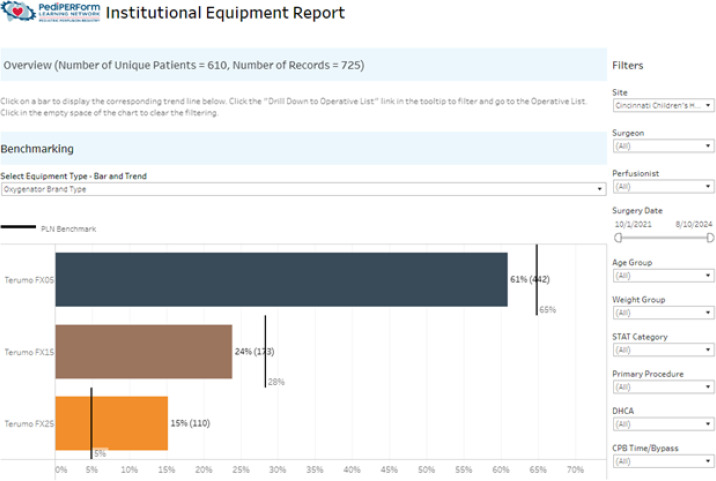
Institutional equipment report showing number and type of oxygenator used at a given institution.

Institutional reports return values for the same measures as found in the blinded site reports (Figure 2). However, this data is specific to the individual institution. In these reports, the center’s clinical champion can drill down to specific cases, facilitating granular data investigation for each participating site. Institutional data is only visible to the specific institution that submitted the record.

**Figure 3:**
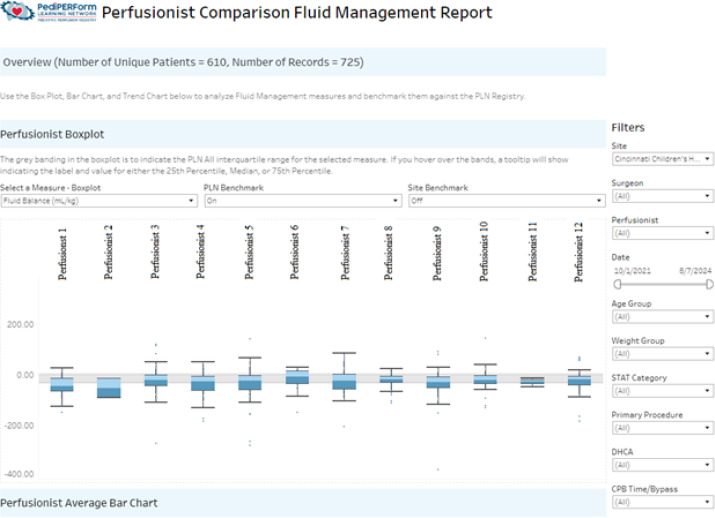
Perfusionist comparison report showing box plot of CPB fluid balance for a list of perfusionists from a given institution.

**Figure 4:**
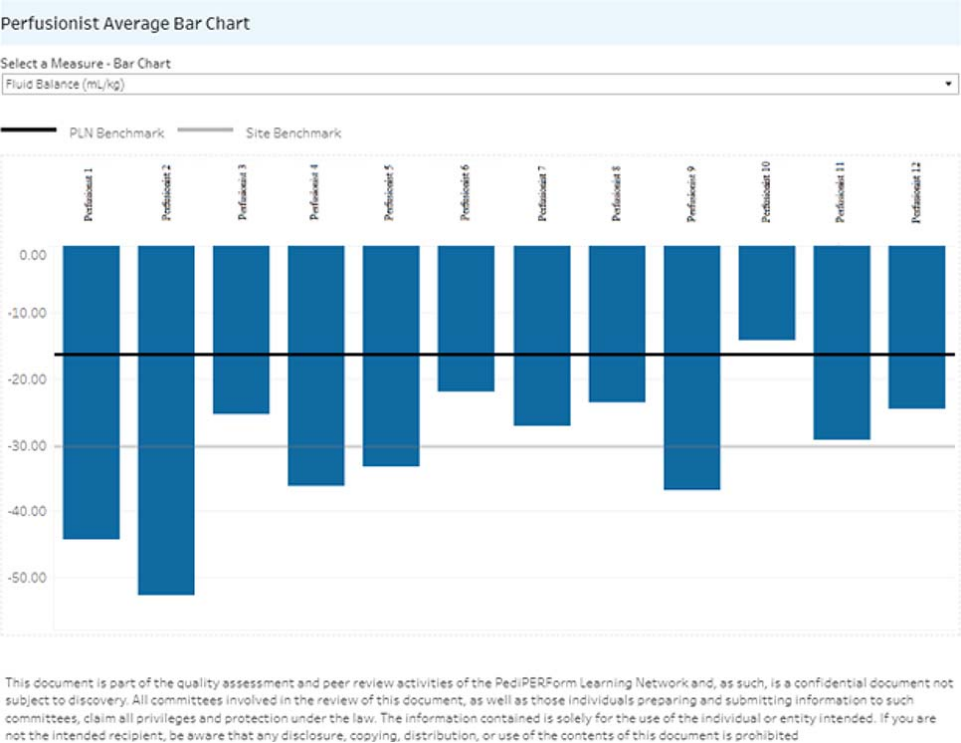
Perfusionist comparison report – Averages showing averages of CPB fluid balance for a list of perfusionists from a given institution.

The perfusionist comparison report, like the institutional report, only provides data for the institution. Data can be broken down to specific perfusionists (Figures 3 and 4). This visualization provides a center with valuable information regarding internal practice variation. Utilizing this data, a perfusion department can improve quality and reduce practice variation within their practice.

Participating in the PLN registry costs above $7,250 at the time of publication. Arbormetrix charges $5,250 annually for the dashboard and the report generating software. PLN charges $2,000 a year which is split to fund a project manager at Cincinnati Children’s Hospital and fund data managers and data storage at the University of Michigan. The costs of the CardioAccess and Lumedx modules are set by those said companies and can be different amongst participating centers. One of the participating centers uses its own internal software and submits directly to Arbormetrix, bypassing the costs of CardioAccess or Lumedx altogether.

## Discussion

Congenital CPB surgeries account for a low percentage of all cardiac cases performed in the United States. With over 5,000 surgical variations to repair congenital defects a range of patient size from less than 1 kg to greater than 100 kg, and an average of 200 procedures being performed in most US centers, the ability to obtain sufficient data to extract meaningful statistical observations is quite difficult. The need to combine congenital heart surgery data has been shown to be beneficial by many registries.

The STS database has been instrumental in defining and enhancing risk adjustment categories and mortality rates by pooling data from nearly all US centers and European registries [7]. The latest data harvest of the STS Congenital Heart Surgeon’s Database includes a four-year period from January 1, 2019, to December 31, 2022 [8]. Besides the enhancements to risk assessments, mortality, risk stratifications, and center size success rates, many other observations and conclusions can be surmised from this set of combined center data.

As an example of how this data can be utilized, Waldman and Ing commented on how anesthesia can use the STS database. They found that operation room tracheal extubation success occurred in children (53%) versus neonates, infants, and adults, and following ASDs (72%) and Fontans (60%) [9]. Early extubation in the operating room is associated with reduced morbidity, length of stay, and improved patient experience [10].

The Advanced Cardiac Therapies Improving Outcomes Network (ACTION) is another widely successful collaborative that combines multicenter data in pediatric heart failure patients from 68 sites to improve outcomes. ACTION assessed 9 years of data from 838 ventricular assist device patients to develop a prognostic tool to predict mortality. Boucek et al. found that identifying risk factors for mortality before VAD implantation, such as TPN, dialysis, and mechanical ventilation, can assist in patient selection for primary mechanical support placement [11]. Hollander utilized ACTION registry data to conclude higher mortality rates in continuous flow devices compared to pulsatile VADs [12].

The PLN will also not limit itself solely to registry publications. October of 2024 will see an inaugural meeting of member centers to discuss registry format, data, future research projects, and, most importantly, the unblinded viewing of center data to promote discussion and clinical practice improvements. As a new registry, the PLN aims to grow its membership in the US and internationally.

In conclusion, the developers of the PLN registry, in partnership with other strategic registries, hope to foster learning and collaboration surrounding the practice of pediatric and congenital perfusion in a grand effort to ultimately improve the care delivered to all patients.

## Data Availability

Data is from the PLN test environment therefore is not available. All fields are available from the corresponding author by request.
